# Nurses’ perceptions of factors influencing treatment engagement among patients with cardiovascular diseases: a systematic review

**DOI:** 10.1186/s12912-021-00765-2

**Published:** 2021-12-20

**Authors:** Amineh Rashidi, Lisa Whitehead, Prachi Kaistha

**Affiliations:** 1 School of Nursing and Midwifery, Edith Cowan University, 270 Joondalup Drive, Joondalup, WA 6027 Australia; 2 Oxford Brookes University, London, UK

**Keywords:** ‘Cardiovascular rehabilitation’, ‘Treatment activation’, ‘Treatment engagement’, ‘Treatment participation’, ‘Cardiovascular disease’, ‘Qualitative study’

## Abstract

**Background:**

Nurses are key to the success of patient engagement, yet we know little about nurses’ perceptions on treatment engagement and how they can contribute to treatment engagement. Qualitative evidence to identify factors that influence treatment engagement among patients with CVD from nurse’s perspective is limited.

**Methods:**

This systematic review of qualitative research was based on the PRISMA reporting guidelines. The Joanna Briggs Institute (JBI) Critical Appraisal Checklist was used to assess quality by two reviewers independently. Data were collected from Medline, Web of Science, CINAHL, PsychINFO, Embase- Non-Medline, Scopus, and the Cochrane Library, were systematically searched from 2001 to 2020. The search strategy included keywords and MeSH terms to identify relevant studies written in English.

**Results:**

Eight articles were included in the review. Four key themes were synthesised from the findings: nurses need training and up to date information, providing support for patients, patient motivation to engage with treatment plans and perceived lack of time.

**Conclusion:**

Nurses described the importance of training to help them support patients to engage as effectively as possible and their role in providing social and psychological support. They also described the importance of patient motivation to engage in a treatment and plan and sustain engagement and time.

**Supplementary Information:**

The online version contains supplementary material available at 10.1186/s12912-021-00765-2.

## Background

Globally, more people die from cardiovascular disease per year compared to any other condition accounting for 31% (17.9 million) in 2016 [1]. Management of CVD is crucial to reduce the risk of disease and further complications. Management follows a standardised pathway, including the use of medication therapy and lifestyle modification [2]. The management of CVD requires people living with CVD to fully engage in their treatment plan and to attend cardiac rehabilitation after a cardiac event [3]. .Nurses play an important role in working with people with cardiac disease to engage them in their disease management [4–6]. The terms involvement and participation are often used synonymously with engagement and a general definition is the ability of patients to manage their health and to adjust management practices as needed [7, 8]. Patient engagement in treatment is associated with improved health outcomes, satisfaction with care and the reduction of health care costs [6, 9, 10]. From a nursing perspective, the ability to engage patients has been related to the ability to spend time with them and provide up to date, evidence-based information [6, 11]. Patient motivation and support have been described as pivotal in engagement with treatment plans [12]. Recent studies [13, 14] indicate that participation in cardiac rehabilitation is low.

Nurses play a significant role in promoting engagement with treatment plans in inpatient and outpatient settings and in cardiac rehabilitation [11, 15]. Nurses can directly facilitate and encourage patient engagement or participation in treatment plans [10] and support sustained engagement [16, 17]. However, little is known about nurses’ perceptions on patient engagement and the reasons why engagement is not always achieved to inform strategies to improve engagement. To date, one systematic review has focused on the process of patient engagement only in cardiac rehabilitation [18] but this did not focus on nurses’ perspectives. Also, no review integrating the findings of qualitative studies designed to explore factors that influence treatment engagement has been conducted. The aim of this review was to synthesise the qualitative literature on nurses’ perspectives of the factors that influence treatment engagement among patients with CVD.

### Aim

To synthesize qualitative evidence on nurses’ perceptions of factors that influence treatment engagement among people diagnosed with CVD.

## Methods

This systematic review was conducted in accordance with the principles of the Preferred Reporting Items for Systematic Reviews and Meta-Analyses: The PRISMA Statement [19]. This review was registered in PROSPERO (CRD42020176543).

### Search strategy

Eight electronic databases MEDLINE (EBSCOhost), CINAHL (EBSCOhost), PsycINFO (EBSCOhost), Cochrane (Ovid), EMBASE (Ovid), Web of Science (Web of Science), Scopus (Scopus) and Joanna Briggs Institute (JBI) (Ovid) were searched for peer reviewed publications in English from January 2001–December 2020. Keywords and Medical Subject Headings (MeSH) Terms were used to identify relevant literature. Key terms relevant to cardiovascular diseases and treatment, engagement, perceptions, and experiences were used (Additional file 1: Appendix 1). These keywords were used as MeSH terms and were also revised for use in the selected database. Advice and support from a subject librarian was sought in the construction of the search strategies.

### Study selection and inclusion criteria

Studies were included if they met the following eligibility criteria: (1) Population: identify as nurses; (2) Phenomena of Interest: perceptions of factors influencing treatment engagement (3) Context: cardiovascular rehabilitation, hospital/healthcare settings in developed countries (3) Study Design: qualitative research. Journal articles published in English between 2001 to 2020 were included. The period of 19 years was established due to the increase in the discourse on treatment engagement and adherence, which began around this time. Treatment engagement refers to commitment to the therapeutic process, an active role in their treatment care, and a therapeutic relationship with the therapist [8, 20–22].

Studies conducted in developing countries, focused on open heart surgery, cardiac procedures, non-ischemic heart failure and studies with a focus on the comorbidity of CVD with mental health conditions were excluded. According to the International Monetary Fund, developed countries are considered to be countries with an advanced economy [23]. Studies conducted in developing countries were excluded, because the program for treatment engagement and health systems in developed countries are unlikely to be directly transferable to developing countries. All citations identified in the searches were exported to EndNote and duplicate records were removed. Screening the titles and abstracts of the full text for their relevance against the inclusion criteria was conducted by two independent reviewers.

### Quality appraisal and data extraction

The quality of included studies was appraised using the JBI Qualitative Assessment and Review Instrument for qualitative studies. Two independent reviewers (the primary and second reviewers) assessed the methodological quality of the included studies. If there were disagreements between the reviewers, a third reviewer was involved to reach consensus. No studies were excluded based on the methodological quality. The qualitative data from the included studies were extracted using the data extraction tool and specific details about the population, context, culture, geographical location, study methods, and the phenomena of interest relevant to the aim and objective were extracted.

### Data synthesis

This review used the JBI approach, meta-aggregation to synthesis qualitative data [24, 25]. The research findings from the included studies were synthesized to create a set of categories that represented aggregation [24, 25]. Each extracted finding was examined based on three levels of evidence: ‘unequivocal’, ‘credible’ or ‘not supported’ [24, 25]. “Unequivocal: findings accompanied by an illustrations beyond a reasonable doubt, therefore not open to challenge, credible: findings accompanied by the illustrations that are plausible and inferred from the date, therefore open to challenge and unsupported: findings not supported by the data [4, 25]. Findings not supported by a quotation were not included in the synthesis. The primary reviewer grouped the findings into categories based on the similarity in meaning and concepts, then aggregated by commonality into synthesized categories. These categories were discussed with the second reviewer and the synthesized findings discussed by the review team until consensus was reached.

### Assessing confidence

The ConQual approach was used to assess confidence in the output of qualitative research synthesis [25]. According to the ConQual approach (Additional file 1: Appendix 2), the dependability and credibility of each study were considered. Dependability of the extracted findings was assessed by 5 questions (Q2, Q3, Q4, Q6 and Q7) of the standardised JBI SUMARI instrument for the Critical Appraisal of Qualitative Evidence. Scores of 4 or 5 out of five suggest a high level of dependability, while scores of 2 or 3 suggest a moderate level of dependability. In this review, seven studies [3, 15, 26–30] were assessed as having a high level of dependability and one study [31] a moderate level of dependability. Credibility was established by assessing the congruency between the author’s interpretation and supporting data. In this review, the findings were a combination of unequivocal and credible, therefore, the overall credibility of the findings were downgraded from a high to moderate level of credibility.

## Results

### Study inclusion

The selection process for inclusion in the systematic review is displayed in Additional file 1: Appendix 3. A total of 2333 records were identified through a systematic search. Duplicates (*n* = 1523) were excluded. Title and abstract screening were conducted for 810 articles. Forty articles underwent full-text screening, and this was conducted by two independent reviewers. Eight articles were retained for quality appraisal and were included in the synthesis.

### Methodological quality of included studies

The assessment of the methodological quality of the included studies is displayed in Additional file 1: Appendix 4. All included qualitative studies indicated congruity between the research methodology and the research question or objectives and utilized appropriate data collection methods and approach to data analysis [3, 15, 26–31]. The cultural or theoretical perspective in relation to the research was discussed in three studies [26–28]. The influence of the researcher on the research and vice-versa was identified in seven studies.

### Characteristics of included studies

Eight qualitative studies were included in the review [3, 15, 26–31]. All studies were conducted in a cardiac care setting either in a hospital or community setting [3, 15, 26–31]. Two studies were conducted in England [15, 30], and one study each in Sweden [31], the Netherlands [29], Ireland [28] Australia [26, 27], and Norway [3]. The sample size ranged from seven [27] to 22 participants [3]. Seven qualitative studies used face-to-face interviews [15, 26–31], one study [3] used focus groups. Additional file 1: Appendix 5 presents an overview of the study characteristics.

### Review findings

Thirty-two findings were extracted and synthesised into four categories (Additional file 1: Appendix 6).

### Nurses need training and up to date information

Findings from five studies [26–30] contributed to this category. Nurses perceived that training and education sessions are important in equipping them with information and skills to establish and engage patients in treatment planning. Nurses felt confident in providing advice or information relating to lifestyle, but they felt that medication was the area about which they would have liked ongoing training to assist patients to more fully engage patients in treatment planning: “*because medication is changing so much, we’ve got to have ongoing training all the time. We haven’t had enough training at the moment” *([30], p.186).

Nurses also believed that nurses who held a mentorship role in cardiac rehabilitation programmes required ongoing training: “*more preparation and training may be needed to adequately prepare mentors for the role. It was actually very hard work especially as you travel the highs and lows with patients as they recover*” ([27], p.96). In particular, less experienced nurses were described as requiring concise and clear information to guide patients in the right direction *“at least for those with less experience that might be unsure about what information they are supposed to give*” ([3], p.5). Nurses believed that knowledge relating to surrounding their role in CVD management and treatment engagement must be updated to nurses to provide accurate medical advice: *“you need up to date knowledge in cardiology to be giving the right advice”* ([28], p.587). Also, nurses perceived that training and coaching sessions could equip them with essential knowledge and skills to enhance patients’ engagement in their treatment plan, through collecting information on symptoms, discussing lifestyle changes, conducting assessments and providing routine follow-up care to maintain change: *“after the training, I felt I had a lot of tools I could apply to patients. I was equipped with a lot of techniques for gaining effects in patients. Now I make it more specific and explore with the patient how to continue*” ([29], p.6). In relation to cardiac rehabilitation programs, nurses found that training and skills workshops improved their knowledge and assisted them to implement a homebased cardiac rehabilitation program: “*being able to adapt the program to suit the individual person, and tailor it to suit the habits and interests of the individual was important*” ([26], p.80).

### Providing support for patients

Findings from five studies [15, 26, 27, 29, 31] informed this category. Being able to provide patients with support was described as a significant factor in engaging patients in their treatment plan. Nurses perceived psychological support as integral to patient recovery and engagement in a treatment plan: “I*’ve got to be honest, I mean, sometimes I’ve left a cardiac rehabilitation clinic and all that we have addressed is the psychological side of things”* ( [15], p.4). Peer support was perceived as an important element in one study [15]. Peer support provided mutual moral support that encouraged patients to engage in their treatment plan. Nurses described benefits of sharing the experience of engaging in cardiac rehabilitation with others: *“Patients get a huge amount of benefit just in talking to each other, and so the problem, the trouble solving, the solutions, “oh I do this and just seeing how other people are getting on, the little supportive networks that they strike up when they’re actually in the waiting room waiting for us to assess them and they’ve already got their own counselling and social network going on there”*([15], p.6). Consultation was also viewed as another form of support [29, 31]. Nurses perceived that consultation with patients prior to discharge could strengthen patient’s beliefs about the feasibility of their engagement in a treatment plan: *“If you would send them home with an activity log but without consultations, then no one would fill it in. You have to make it specific; otherwise, it won’t work”* ([29], p.7). One study [27] noted that a mentor was another form of support and through facilitation helped patients to engage in their treatment plan. The provision of timely support and guidance for patients after hospital discharge was described as playing a significant role in assisting patient recovery and emotional adjustment. Nurses also perceived that it was important to patients that they possessed a level of empathy: “*empathy* (*for the patient) is very important and an understanding of what it’s like for patient’s to experience a life-changing event* ([27] , p.97). Mentorship was described as reinforcing healthy behaviour and kept patients focused and motivated: “*mentors can give patients hope and motivation to change poor lifestyle choices that may have impacted on their illness*” ([27], p.97). Nurse mentors could help patients to learn about their illness, address knowledge gaps and improve understanding of the benefits of engaging in their treatment plan *“sound knowledge of cardiac rehab principles and cardiac risk factors, plenty of life skills and a large kit bag of heart health knowledge are needed to cater for individual patient”. Patient misconceptions about coronary heart disease need to be corrected before they can learn to move forward and adopt the central role in their own health”* ([26], p.80).

### Patient motivation to engage with treatment plans

Four studies contributed to this category [3, 26, 27, 29]. Nurses perceived that their contribution to the engagement of patients’ in their treatment plan was a primary part of their role. They believed that a lack of motivation can negatively impact on patient engagement. Nurses described engaging poorly motivated patients as difficult and they sometimes felt responsible: *“I felt a feeling of frustration and failure when the person involved was unable to successfully make changes to their lifestyle”* ([27], p.98). Nurses perceived motivating patients to engage in their treatment plan as a challenge. They believed that the use of tools could help them to encourage patients to enhance physical activity: “*the main reason was that it’s difficult to motivate people to increase their physical activity. I could use some tools for how I could handle this the best way*” ([29], p.5). Nurses also perceived that patient engagement depended on patients’ motivation and willingness to engage coupled with commitment to attain goals. When these were not evident, nurses questioned their efforts to engage patients: “*for me, it’s more fun to support a motivated patient who does his homework perfectly compared to a patient who brings a completely empty diary. Then, you think this costs me forty-five minutes, and that patient actually does not do anything. It’s a lot more fun when they say, ‘I deliberately went cycling to reach my goal.’ Yes, then you really feel like that’s what I am doing it for*” ([29], p.6). Nurses believed that motivation is crucial for patient engagement in cardiac rehabilitation “*we cannot make changes if the patients do not take part in it*” ([3], p.1612). Nurses perceived that information related to the illness, symptoms management, medication and dietary information, lifestyle factors and physical activity is necessary to understand the patient needs. One study [29] reported that nurses expressed a need to enhance their skills to increase patient’s motivation in relation to physical activity: “*the main reason was that it’s difficult to motivate people to increase their physical activity. Very often, questions about patients’ motivation remain superficial, and I wanted to know how I am going to ask in-depth questions about their motivation?”* ( [29], p.5).

### Perceived lack of time

The perception that nurses experienced a lack of time was described in two studies [28, 31]. The need for more time during follow up appointments to explore patients’ understanding of their illness and their concerns about treatment was reported: “*the risk of there being a lack of time during follow-up visits if the visit took a bit longer than usual and the risk that there was no time for preparation on their side. In line with this, professionals brought up the issue that they did not have enough time to log on and check the values of patients’ self-reported data”* ( [31], p.473). Nurses perceived that there was not enough time to engage patients in the development of their treatment plan as part of a health promotion strategy. Therefore, time constraints sometimes impacted on nurse’s ability to provide a quality service: “a lot of the time we don’t get to see patients unless they have a clinical nursing need, and if we do there is no time for health promotion, that can’t be effective” ( [28], p. 587).

## Discussion

The purpose of this review was to explore nurse’s perceptions about the factors that influence treatment engagement among patients with cardiovascular disease. These factors include perceived lack of time, patient motivation to engage with treatment plans, providing support for patients and nurses need training and up to date information.

The findings of the present review suggests that sufficient training could facilitate nurses to acquire knowledge and skills and to incorporate these skills to support patients to self-manage through advice and to enhance patient’s engagement in their treatment plan. The findings also indicated that training is important in increasing nurses’ skills and knowledge to assess and identify patients who are at risk of disengagement and to enhance their ability to promote patient engagement [12]. The need for support was the most frequently described factor. Ongoing support from nurses was identified as important in embedding treatment plans and to continued engagement post discharge in physical activity, taking medication and making dietary changes [32, 33]. According to our findings, peer support was also described as a support that can lead to positive outcomes. However, the role of peer support has not been sufficiently studied and is an area that could be harnessed further to encourage patients to engage in cardiac rehabilitation [34].

Based on the results of our review, empathy among nurses was found to be related to treatment engagement. Understanding the patient experience during recovery can be related to the facilitation of positive health outcomes by both identifying patients’ needs and increasing treatment engagement. Parallel to our study, the study by Martin et al. [35] showed that empathic understanding of the patient’s perspective could improve treatment engagement among patients and this is an area for further study. However, further work needs to be considered to understand how nurses can support patients over time and promote sustained behaviour change.

We found that the concept of motivation was used by nurses to facilitate healthy behaviour by reinforcing patients’ self-efficacy or confidence. Interestingly, we found that nurses agreed that motivation was ultimately responsible in the development of specific behavioural tools and strategies needed to perform health-related behaviours in turn related to engaging in and maintaining treatment and this findings is supported in the wider literature [36]. The findings are also in line with a study in which surveillance and observation were found to support motivation for positive change in patient behaviours and subsequently encouraging patients to commit to cardiac rehabilitation [37]. Based on our findings, without motivation, nurses could have challenges to empower patients to positively influence their level of health. Lack of time was reported as a limiting factor during follow up or recovery visits. Further studies have indicated that insufficient time with the patient inhibited the establishment of therapeutic relationships that are necessary for patient engagement [10, 38]. A longitudinal study of nursing care reported that it is important to allow enough time to spend with patients in order to understand and address patients’ needs and to plan and provide good quality care and follow-up [11].

A major strength of this review was the methodological rigour with the review in accordance with the JBI guidelines. However, this systematic review has some limitations. Firstly, the exclusion of non-English studies in this review may not confidently capture the essence of perceptions of nurses in non-English speaking cultures. Secondly, due to homogeneity in study characteristics, including the target population and healthcare setting, findings may not be generalizable beyond nurses working in cardiac settings. Thirdly, in this review, only studies reporting nurses’ perspective were included. Further research examining the perspective of other health care providers is needed to gain a more comprehensive and holistic view of this topic.

## Conclusion and implications

The importance of the role of nurses in promoting treatment engagement is well supported nationally and internationally. The findings of this review described the factors that influence treatment engagement in treatment plans among people living with CVD from a nursing perspective. Nurses perceived that training, providing support, patient motivation and patient spending time with patients could enhance treatment engagement. Reflection on their own practice could provide an opportunity for nurses to formulise more standardised approaches to engaging patients. Nurses need to comprehensively assess patient motivation and patients’ needs in order to tailor an approach to engage patients in their treatment plan. This review provides new insights concerning the perceptions of nurses delivering a home-based cardiac rehabilitation program to CVD patients post hospital discharge. The implications for practice merit further study to contribute to the development of acceptable and effective treatment engagement strategies.

## Supplementary Information

**Additional file 1.**

## Data Availability

All data generated or analysed during this study are included in this published article [and its supplementary information file 2].
