# Supplementary material for: Nurses’ perceptions of factors influencing treatment engagement among patients with cardiovascular diseases: a systematic review
Source: BMC Nurs. 2021 Dec 20;20:251. doi: 10.1186/s12912-021-00765-2 (PMC8686541; doi:10.1186/s12912-021-00765-2)
Supplement: Supplementary file 1 — Additional file 1. [file 12912_2021_765_MOESM1_ESM.docx]

**Appendices**

**Appendix 1:**

**Medline search strategy (Filtered for January 2001-December 2020 and English)**

("cardiovascular disease"" OR (MH "Cardiovascular Diseases")) OR (""coronary artery disease*"" OR (MH "Coronary Artery Disease") OR (MH "Coronary Disease")) OR (""coronary heart disease*"") OR ((MH "Acute Coronary Syndrome") OR ""acute coronary syndrome"") OR ((MH "Myocardial Infarction") OR ""myocardial infarct*"") OR ((MH "Angina, Unstable") OR ""unstable angina"") OR ((MH "Myocardial Ischemia") OR ""myocardial ischemia"") OR (""ischemic heart failure"" OR (MH "Heart Failure")) OR "CVD" OR "CAD" OR "MI" OR "CHD" OR ((MH "Heart Arrest") OR ""cardiac arrest"") OR (""heart disease"") OR (""heart attack"") OR ((MH "Heart Diseases")) OR "(MM “coronary Stenosis”) MH “ hypertension +DH/DT RH/NU” AND “perception*” AND “ Treatment engagement” OR Treatment Involvement” OR “Treatment Participation” AND “Nurse*” AND ("factor" OR "reason*" OR "influence*" OR "cause*") AND (MM “Medication Therapy Management” ) OR (MM “life Style”) (MM “Sedentary Lifestyle”) OR (MM “Exercise”) OR (MM “Motor Activity”) AND “Cardiac Rehabilitation” (MM “Diet”) (MM “Diet, Fat-Restricted”) OR (MM “Diet, Carbohydrate-Restricted”) OR (MM “Diet, Soduim-Restricted”)

**CINAHL: (Filtered for 2001-2020 and English)**

("cardiovascular disease"" OR (MH "Cardiovascular Diseases")) OR (""coronary artery disease*"" OR (MH "Coronary Artery Disease") OR (MH "Coronary Disease")) OR (""coronary heart disease*"") OR ((MH "Acute Coronary Syndrome") OR ""acute coronary syndrome"") OR ((MH "Myocardial Infarction") OR ""myocardial infarct*"") OR ((MH "Angina, Unstable") OR ""unstable angina"") OR ((MH "Myocardial Ischemia") OR ""myocardial ischemia"") OR (""ischemic heart failure"" OR (MH "Heart Failure")) OR "CVD" OR "CAD" OR "MI" OR "CHD" OR ((MH "Heart Arrest") OR ""cardiac arrest"") OR (""heart disease"") OR (""heart attack"") OR ((MH "Heart Diseases")) OR "(MM “coronary Stenosis”) MH “ hypertension +DH/DT RH/NU” AND “perception*” AND “ Treatment engagement” OR Treatment Involvement” OR “Treatment Participation” AND “Nurse*” AND ("factor" OR "reason*" OR "influence*" OR "cause*") AND (MM “Medication Therapy Management” ) OR (MM “life Style”) (MM “Sedentary Lifestyle”) OR (MM “Exercise”) OR (MM “Motor Activity”) AND “Cardiac Rehabilitation” (MM “Diet”) (MM “Diet, Fat-Restricted”) OR (MM “Diet, Carbohydrate-Restricted”) OR (MM “Diet, Soduim-Restricted”)

**PsycINFO: (Filtered for 2001-2020 and English)**

("cardiovascular disease"" OR (MH "Cardiovascular Diseases")) OR (""coronary artery disease*"" OR (MH "Coronary Artery Disease") OR (MH "Coronary Disease")) OR (""coronary heart disease*"") OR ((MH "Acute Coronary Syndrome") OR ""acute coronary syndrome"") OR ((MH "Myocardial Infarction") OR ""myocardial infarct*"") OR ((MH "Angina, Unstable") OR ""unstable angina"") OR ((MH "Myocardial Ischemia") OR ""myocardial ischemia"") OR (""ischemic heart failure"" OR (MH "Heart Failure")) OR "CVD" OR "CAD" OR "MI" OR "CHD" OR ((MH "Heart Arrest") OR ""cardiac arrest"") OR (""heart disease"") OR (""heart attack"") OR ((MH "Heart Diseases")) OR "(MM “coronary Stenosis”) MH “ hypertension +DH/DT RH/NU” AND “perception*” AND “ Treatment engagement” OR Treatment Involvement” OR “Treatment Participation” AND “Nurse*” AND ("factor" OR "reason*" OR "influence*" OR "cause*") AND (MM “Medication Therapy Management” ) OR (MM “life Style”) (MM “Sedentary Lifestyle”) OR (MM “Exercise”) OR (MM “Motor Activity”) AND “Cardiac Rehabilitation” (MM “Diet”) (MM “Diet, Fat-Restricted”) OR (MM “Diet, Carbohydrate-Restricted”) OR (MM “Diet, Soduim-Restricted”)

**Cochrane: (Filtered for 2001-2020)**

("cardiovascular disease" OR "coronary artery disease*"" OR "Coronary Disease" OR"coronary heart disease*"OR "Acute Coronary Syndrome" OR "Myocardial Infarction"myocardial infarct*"OR "Angina, Unstable" OR "unstable angina"OR "Myocardial Ischemia" OR "ischemic heart failure"" OR "Heart Failure" OR "CVD" OR "CAD" OR "MI" OR "CHD" OR "Heart Arrest" OR "cardiac arrest"OR "heart disease" OR "heart attack" OR “coronary Stenosis” OR” hypertension” AND “(perception*”) AND (“Treatment engagement” OR Treatment Involvement” OR “Treatment Participation”) AND (“Nurse*” )AND ("factor" OR "reason*" OR "influence*" OR "cause*") AND ( “Medication Therapy Management” OR “life Style” OR “Sedentary Lifestyle” OR “Exercise” OR “Motor Activity” AND (“Cardiac Rehabilitation” OR “Diet” OR “Diet, Fat-Restricted” OR “Diet, Carbohydrate-Restricted” OR “Diet, Soduim-Restricted”)

**WoS: (Filtered for 2001-2020 and English)**

("acute coronary syndrome" OR "myocardial infarct*" OR "cardiovascular disease" OR "coronary artery disease*" OR "coronary heart disease*" OR "unstable angina" OR "myocardial ischemia" OR "ischemic heart failure" OR " CVD" OR "CAD" OR "ACS" OR "MI" OR "CHD" OR "heart attack" OR "cardiac arrest" OR "heart disease*") AND ("factor*" OR "cause*" OR "reason*" OR "influence") AND (treatment engagement) OR (treatment involvement) OR (treatment participation) OR # 1OR # 2 OR # 3 AND ( nurse*) AND (perception*) AND (healthy lifestyle) AND (healthy diet) AND (medication*) OR (medication management) AND ( exercise) OR (physical activity).

**Embase**

('cardiovascular disease'/exp OR 'acute coronary syndrome'/exp OR 'heart infarction'/exp OR 'coronary artery disease'/exp OR 'ischemic heart disease'/exp OR 'coronary heart disease' OR 'myocardial infarction' OR 'unstable angina pectoris'/exp OR 'heart muscle ischemia'/exp OR 'ischemic heart failure'/exp OR cvd OR cad OR acs OR mi OR chd OR 'heart attack' OR 'heart disease'/exp OR 'heart arrest'/exp) AND (factors OR influence OR cause OR reason) AND (treatment engagement OR treatment involvement OR treatment participation) AND ( nurses) AND (perceptions) AND (healthy lifestyle) AND (healthy diet) AND (medications) AND ( exercise OR physical activity) AND (2001:py OR 2002:py OR 2003:py OR 2004:py OR 2005:py OR 2006:py OR 2007:py OR 2008:py OR 2009:py OR 2010:py OR 2011:py OR 2012:py OR 2013:py OR 2014:py OR 2015:py OR 2016:py OR 2017:py OR 2018:py OR 2019:py OR 2020:py) AND [humans]/lim AND [english]/lim

**Scopus**

( ( TITLE-ABS-KEY ( " treatment engagement " ) ) OR ( TITLE-ABS-KEY ( " treatment involvement " ) ) OR ( TITLE-ABS-KEY ( treatment participation * ) ) AND ( ( TITLE-ABS-KEY ( "perception*" ) ) AND ( ( TITLE-ABS-KEY ( "nurse*" ) ) AND ( ( TITLE-ABS-KEY ( " healthy lifestyle " ) ) OR ( TITLE-ABS-KEY ( "healthy diet" ) ) AND ( ( TITLE-ABS-KEY ( " exercise " ) ) OR ( TITLE-ABS-KEY ( "physical activity " ) ) AND ( ( TITLE-ABS-KEY ( " medication* " ) ) OR ( TITLE-ABS-KEY ( "medication management”) AND ( ( TITLE-ABS-KEY ( "influence*" ) ) OR ( TITLE-ABS-KEY ( "reason*" ) ) OR ( TITLE-ABS-KEY ( cause* ) ) OR ( TITLE-ABS-KEY ( factor* ) ) ) AND ( ( TITLE-ABS-KEY ( "cardiovascular disease" ) ) OR ( TITLE-ABS-KEY ( "coronary heart disease*" ) ) OR ( TITLE-ABS-KEY ( "coronary artery disease*" ) ) OR ( TITLE-ABS-KEY ( "acute coronary syndrome" ) ) OR ( TITLE-ABS-KEY ( "myocardial infarct*" ) ) OR ( TITLE-ABS-KEY ( "unstable angina" ) ) OR ( TITLE-ABS-KEY ( "myocardial ischemia" ) ) OR ( TITLE-ABS-KEY ( "ischemic heart failure" ) ) OR ( TITLE-ABS-KEY ( cvd ) ) OR ( TITLE-ABS-KEY ( cad ) ) OR ( TITLE-ABS-KEY ( acs ) ) OR ( TITLE-ABS-KEY ( mi ) ) OR ( TITLE-ABS-KEY ( chd ) ) OR ( TITLE-ABS-KEY ( "heart disease*" ) ) OR ( TITLE-ABS-KEY ( "heart attack" ) ) OR ( TITLE-ABS-KEY ( "cardiac arrest" ) ) ) AND ( LIMIT-TO ( PUBYEAR , 2020 ) OR LIMIT-TO ( PUBYEAR , 2019 ) OR LIMIT-TO ( PUBYEAR , 2018 ) OR LIMIT-TO ( PUBYEAR , 2017 ) OR LIMIT-TO ( PUBYEAR , 2016 ) OR LIMIT-TO ( PUBYEAR , 2015 ) OR LIMIT-TO ( PUBYEAR , 2014 ) OR LIMIT-TO ( PUBYEAR , 2013 ) OR LIMIT-TO ( PUBYEAR , 2012 ) OR LIMIT-TO ( PUBYEAR , 2011 ) OR LIMIT-TO ( PUBYEAR , 2010 ) OR LIMIT-TO ( PUBYEAR , 2009 ) OR LIMIT-TO ( PUBYEAR , 2008 ) OR LIMIT-TO ( PUBYEAR , 2007 ) OR LIMIT-TO ( PUBYEAR , 2006 ) OR LIMIT-TO ( PUBYEAR , 2005 ) OR LIMIT-TO ( PUBYEAR , 2004 ) OR LIMIT-TO ( PUBYEAR , 2003 ) OR LIMIT-TO ( PUBYEAR , 2002 ) OR LIMIT-TO ( PUBYEAR , 2001 ) ) AND ( LIMIT-TO ( LANGUAGE , "English" ) )

**JBI**

(“acute coronary syndrome” OR “heart disease” OR “myocardial infarction” OR "cardiovascular disease") AND AND “(perception*”) AND (“Nurse*” )AND ("factor" OR "reason*" OR "influence*" OR "cause*") AND ("factor*" OR "cause*" OR "reason*" OR "influence") AND (“treatment engagement” OR “treatment involvement” OR “treatment participation”.

**Appendix 2: Dependability scores for included studies**

| Citation | Is there congruity between the research methodology and the research question or objectives | Is there congruity between the research methodology  and the methods used to collect data? | Is there congruity between the research methodology and the representation and analysis of data? | Is there a statement locating the researcher culturally or theoretically? | Is the influence of the researcher on the research, and vice-versa, addressed? | Dependability Score |
| --- | --- | --- | --- | --- | --- | --- |
| Bårdsgjerde et al (2020) | Y | Y | Y | N | Y | 4/5 High |
| Frohmader et al. (2018) | Y | Y | Y | Y | Y | 5/5 High |
| Frohmader et al. (2017) | Y | Y | Y | Y | Y | 5/5 High |
| Hallberg et al (2018) | Y | Y | Y | N | N | 3/5 Mod |
| Heery et al (2018) | Y | Y | Y | Y | Y | 5/5 High |
| Turner et al. (2017) | Y | Y | Y | N | Y | 4/5 High |
| Westland et al. (2018) | Y | Y | Y | N | Y | 4/5 High |
| Wright et al (2001) | Y | Y | Y | U | Y | 4/5 High |

**Appendix 3:** **Study Selection and PRISMA flow diagram (1)**

Identification

Number of additional records identified through other sources (N=0)

Number of records identified through a systematic search (N=2333)

Duplicates removed

(N=1523)

Screening

Records screened through title and abstract (N=810)

Records excluded based on title and abstract (N=770)

Number of full-text articles assessed for eligibility (N=40)

Number of articles excluded on reading full-text (N=32)

Eligibility

Number of articles excluded on critical appraisal (N=0)

Number of articles assessed for quality (N=8)

Included

Number of articles included

**(N=8)**

**Appendix 4: Quality assessment of the included qualitative studies**

| Citation | Q1 | Q2 | Q3 | Q4 | Q5 | Q6 | Q7 | Q8 | Q9 | Q10 | Score /10 |
| --- | --- | --- | --- | --- | --- | --- | --- | --- | --- | --- | --- |
| Bårdsgjerde et al (2020) | Y | Y | Y | Y | Y | N | Y | Y | Y | Y | 9/10 |
| Frohmader et al. (2018) | Y | Y | Y | Y | Y | Y | Y | Y | Y | Y | 10/10 |
| Frohmader et al. (2017) | Y | Y | Y | Y | Y | Y | Y | Y | Y | Y | 10/10 |
| Hallberg et al (2018) | U | Y | Y | Y | Y | N | N | Y | Y | Y | 7/10 |
| Heery et al (2018) | Y | Y | Y | Y | Y | Y | Y | Y | Y | Y | 9/10 |
| Turner et al. (2017) | Y | Y | Y | Y | Y | N | Y | Y | Y | Y | 9/10 |
| Westland et al. (2018) | Y | Y | Y | Y | Y | N | Y | Y | Y | Y | 9/10 |
| Wright et al (2001) | N | Y | Y | Y | Y | U | Y | Y | N | Y | 7/10 |

Y, yes; N, no; U, unclear.

Questions:

1. Is there congruity between the stated philosophical perspective and the research methodology?

2. Is there congruity between the research methodology and the research question or objectives?

3. Is there congruity between the research methodology and the methods used to collect data?

4. Is there congruity between the research methodology and the representation and analysis of the data?

5. Is there congruity between the research methodology and the interpretation of the results?

6. Is there a statement locating the researcher culturally or theoretically?

7. Is the influence of the researcher on the research, and vice-versa, addressed?

8. Are participants, and their voices, adequately represented?

9. Is the research ethical, according to current criteria, or for recent studies, and is there evidence of ethical approval by an appropriate body?

10. Do the conclusions drawn in the research report flow from the analysis, or interpretation, of the data?

**Appendix 5:** **Characteristics of included studies for methodological review**

| **Study** | **Methods for data collection and analysis** | **Country** | **Phenomena of interest** | **Setting/context/culture** | **Participant characteristics and sample size** | **Description of main results** |
| --- | --- | --- | --- | --- | --- | --- |
| Bårdsgjerde et al (2020) | Five focus groups were conducted at two hospitals, one with and one without  percutaneous coronary intervention facilities, between February–November  2018. Participants were recruited through purposive sampling. Twenty-two nurses  experienced in cardiac care participated. The analysis had a hermeneutical approach | Norway | To explore nurses' perceptions of patient participation in different phases of the  myocardial infarction pathway**.** | Hospital | Twenty-two nurses | The findings revealed nurses' perceptions of patient participation in different  phases of the myocardial infarction pathway. Four themes were identified: (a)  variation between paternalism and autonomy in the acute phase; (b) individualization  of dialogue and patient participation during treatment; (c) lack of coherence in  the pathway hinders patient participation at discharge; and (d) cardiac rehabilitation  promotes patients' autonomous decisions in lifestyle changes. |
| Frohmader et al. (2018) | Data were collected from patient telephone interviews in their homes at program completion. An open ended mentor survey was conducted by email and mentor clinical patient notes were recorded by mentors during the six week program (in and out of hospital).Thematic analysis of the three sets of data separately was conducted. | Australia | Patient with acute coronary syndrome (ACS) and mentor perceptions of the impact of the Aussie Heart Guide Program in assisting patient recovery from ACS. | coronary care units | n=13 (7 men and 6 women, aged 35–75 years) and 7 nurses | Mentor perceptions concerning the structures of the home-based CR program included the timely recruitment of patients, mentor training to operationalise the program, commitment to development of the mentor role, and the acquisition of knowledge and skills about cognitive behavioural therapy and patient centred care. Processes included the therapeutic relationship between mentors and patients, suitability of the program and the promotion of healthier lifestyle behaviours. Outcomes identified that patients were satisfied with the program’s audiovisual resources, and the level of support and guidance provided by their nurse mentors. Mentors believed that the program was easy to use in terms of its delivery. |
| Hallberg et al (2018) | Face-to-face interviews, held with 20 patients and 7 health professionals were conducted. . Qualitative thematic analysis was used | Sweden | Views of CVD patients (already taking multiple cardiovascular medicines) on a polypill that could reduce the number of tablets they would need to take. | Primary health care Setting | N=7 health professionals all females aged 33–65 and n= 50 patients , 11 women and 39 men | The self-reporting of BP, symptoms, medication use, medication side effects, lifestyle and well-being was perceived to offer insight into how daily life activities influenced BP and helped motivate a healthy lifestyle. Taking increased responsibility as a patient, by understanding factors affecting one’s well-being, was reported as an enabling factor for a more effective care. Based on the experiences, some challenges were mentioned: for adoption of the system into clinical practice, professionals’ educational role should be extended and there should be a reorganization of care to fully benefit from technology. The patients and professionals gave examples of further improvements to the system, for example, related to the visualization of graphs from self-reports and an integration of the system into the general technical infrastructure. These challenges are important on the path to accomplishing adoption. |
| Heery et al (2018) | This qualitative cross-sectional analysis involved face-to-face, semi-structured interviews with a purposeful  sample of 17 public health nurses. Interviews were audio-recorded, transcribed, subjected to thematic content analysis  and subsequently reported incorporating verbatim quotes. | Ireland | On the phenomenon directly from public health  nurses’ encounters within the context of the current health service. | cardiac-care setting | 17 participants (nurses) were  Female | A significant gap exists between evidence-based guidelines for cardiovascular disease prevention and current  practices. Variations in public health nurses’ training, experience and knowledge result in inconsistent practices, and public  health nurses feel this is specialised area for which they are not equipped. The changing public health nurse role and increasing  workloads result in prioritisation of other nursing duties over health promotion. Ineffective systems for care delivery and a  lack of community-based rehabilitation programmes also negatively impact on secondary prevention practices |
| Turner et al. (2017) | In-depth interviews analysed thematically.  Interviews were held with patients and nurses. | England | To explore patients’ and nurses’ views on the  feasibility and acceptability of providing psychological care  within cardiac rehabilitation services | Cardiac services based in the South West of  England and the East Midlands, UK | 18 patients (all male) and 7 (all female) cardiac nurses. | Patients and nurses viewed psychological support  as central to good cardiac rehabilitation. Patients’ accounts  highlighted the significant and immediate adverse effect  a cardiac event can have on an individual’s mental wellbeing.  They also showed that patients valued nurses  attending to both their mental and physical health, and felt  this was essential to their overall recovery. Nurses were  committed to providing psychological support, believed it  benefited patients, and advocated for this support to be  delivered within cardiac rehabilitation programmes rather  than within a parallel healthcare service. However, nurses  were time-constrained and found it challenging to provide  psychological care within their existing workloads. |
| Westland et al. (2018) | A qualitative study nested within a cluster-randomised controlled trial using semistructured interviews was conducted and thematically analysed. | Netherlands | To evaluate nurses’  perceptions towards the delivery and feasibility of the Activate intervention | Primary care setting | The study sample consisted of 14 primary care nurses | Three key themes emerged concerning nurses’ perceptions of delivering the intervention: nurses’  engagement towards delivering the intervention; acquiring knowledge and skills; and dealing with adherence to  the consultation structure. Three key themes were identified concerning the feasibility of the intervention:  expectations towards the use of the intervention in routine practice; perceptions towards the feasibility of the  training programme; and enabling personal development. |
| Wright et al (2001)) | qualitative study, audio-taped interviews and thematically analysed. | England | To explore what happened  during a patient's initial assessment for secondary prevention  of ischaemic heart disease with a practice nurse  and investigated patients' and practice nurses' views of  nurse-led clinics in primary care. | practice nurse-led care setting | The nurses  ranged in age from 38 to 61 years and 7 Nurses | Nurses were effective in history taking and offering reassurance and dietary  advice, yet were less con®dent in discussing patients' understandings of heart  disease and related medication.  · Practice nurse-led coronary preventive care is acceptable to both nurses and  patients.  · Further practice nurse education is required in heart disease, cardiac  medications and skills necessary for exploring and challenging patients'  understandings of these issues. |

**Appendix 6:** **Themes**

| Synthesised Finding | | | | |
| --- | --- | --- | --- | --- |
| Studies | **Nurses need training and up to date information** | **Providing support for patients** | **Patient motivation to engage with treatment plans** | **Perceived Lack of time** |
| Bårdsgjerde et al (2020) | 🗸 |  | 🗸 | 🗸 |
| Frohmader et al. (2018) | 🗸 | 🗸 | 🗸 |  |
| Frohmader et al. (2017) | 🗸 | 🗸 | 🗸 |  |
| Hallberg et al (2018) |  | 🗸 |  | 🗸 |
| Heery et al (2018) | 🗸 |  |  | 🗸 |
| Turner et al. (2017) |  | 🗸 |  |  |
| Westeland et al. (2018) | 🗸 | 🗸 | 🗸 |  |
| Wright et al (2001) | 🗸 |  |  |  |

1. Moher D, Liberati A, Tetzlaff J, Altman DG. Preferred reporting items for systematic reviews and meta-analyses: the PRISMA statement. Annals of internal medicine. 2009;151(4):264-9.
